# The rate of DNA synthesis in ovaries, fat body cells, and pericardial cells of the bumblebee (*Bombus terrestris*) depends on the stage of ovarian maturation

**DOI:** 10.3389/fphys.2023.1034584

**Published:** 2023-04-11

**Authors:** Radka Zavodska, Hana Sehadova

**Affiliations:** ^1^ Institute of Entomology, Biology Centre CAS, Ceske Budejovice, Czechia; ^2^ Faculty of Education, University of South Bohemia in Ceske Budejovice, Ceske Budejovice, Czechia; ^3^ Faculty of Science, University of South Bohemia in Ceske Budejovice, Ceske Budejovice, Czechia

**Keywords:** *Bombus terrestris*, ovaries, endopolypoidic nurse cells, DNA endoreplication, fat body, pericardial cells, EdU detection

## Abstract

Bumblebees are important pollinators of plants worldwide and they are kept for commercial pollination. By studying the process of oogenesis, we can understand their ontogenetic developmental strategy and reproduction. We describe the anatomy of the ovary of the bumblebee *Bombus terrestris* using 3D reconstruction by confocal microscopy. We found that an oocyte is accompanied by 63 endopolyploidy nurse cells. The number of nurse cells nuclei decreased during oogenesis and the cells are finally absorbed by the oocyte. We monitored the rate of DNA synthesis *in vivo* during 12 h in ovaries, fat body, and pericardial cells in *B. terrestris* queens and workers of different ages. The DNA replication activity was detected on the basis of visualization of incorporated 5-ethynyl-2′-deoxyuridine. DNA synthesis detected in differentiated nurse cells indicated endoreplication of nuclei. The dynamics of mitotic activity varied among different ages and statuses of queens. In 3- to 8-day-old virgin queens, intense mitotic activity was observed in all tissue types investigated. This might be related to the initial phase of oogenesis and the development of the hepato-nephrotic system. In 15- to 20-day-old mated pre-diapause queens, DNA synthesis was exclusively observed in the ovaries, particularly in the germarium and the anterior part of the vitellarium. In 1-year-old queens, replication occurred only in the peritoneal sheath of ovaries and in several cells of the fat body. The similar DNA synthesis patterns in the ovaries of mated pre-diapause queens, ovipositing workers, and non-egg-laying workers show that mitotic activity is related not only to age but also to the stage of ovarian maturation and is relatively independent of caste affiliation.

## 1 Introduction

Currently, more than 200 known species of bumblebees that are important pollinators of crops exist (Velthuis and van Doorn, 2006). They are particularly suitable for pollination in enclosed spaces (greenhouses, *etc.*) where they are not stressed compared to bees. Another advantage for commercial pollination is that they are less aggressive than honeybees and can fly even at relatively low temperatures (6°C) and under cloudy skies (Buchmann and Hurley, 1978). Their greatest economic benefit is for the world production of tomatoes and peppers, where they have a positive effect on fruit quality (more regular shape and higher weight) (Banda and Paxon, 1991). *B. terrestris* as a major reared species is a suitable model organism for studying the physiology and behavior of social insects (Williams, 1998; Baer, 2003; Alaux et al., 2005). However, it cannot achieve the advanced eusocial behavior observed in honeybee colonies. *Bombus terrestris* lives in annual colonies formed by a queen, which mates only once before winter diapause (Duchateau and Velthuis, 1988). The queen fertilizes the eggs with sperm from the spermatheca, and these eggs hatch into workers that are subfertile and are responsible for foraging and brooding (Duncan et al., 2016). At the later stages of colony life, the queen switches to lay unfertilized eggs that emerge into drones. In workers, ovaries begin to develop within 10 days after emerging; however, oocytes mature only in 40% of them, while in the other workers, oocytes remain in the intermediate phase (Duchateau and Velthuis, 1989). Ovarian development in the workers does not affect the process of the queen laying diploid or haploid eggs, and this switch of egg laying has no effects on the oviposition of workers (Duchateau and Velthuis, 1989). However, the presence of the queen slightly delays the ovarian activity of the workers. When workers start laying unfertilized eggs, they compete with the queen for drone production, but the queen gives rise to 95% drones (Alaux et al., 2004; Amsalem et al., 2015). Simultaneously, egg-laying workers inhibit oogenesis in other young workers (Honk et al., 1981a; Bloch and Hefetz, 1999; Foster et al., 2004; Alaux et al., 2006). This competition leads to aggressive behavior of egg-laying workers against the queen and to food shortages that can cause the colony to collapse (Vandoorn and Heringa, 1986; Amsalem and Hefetz, 2011; Gosterit, 2011; Huth-Schwarz et al., 2011; Gosterit et al., 2016).

The reproductive processes of bumblebees may be better understood by a detailed study of oogenesis. Insect oogenesis, i.e., the division, growth, and maturation of oocytes, takes place in ovarioles that form paired ovaries. Within order Hymenoptera, the process of oogenesis has been mainly studied in two representatives of social insects, *B. terrestris* and honeybee *Apis mellifera*. Both species have a typical meroistic polytropic arrangement, where each oocyte is followed by nurse cells (trophocytes). An oocyte and group of nurse cells are enveloped by a layer by follicle cells, which form an egg chamber (Hopkins and King, 1966; Duchateau and Velthuis, 1989; Gutzeit et al., 1993; Dearden, 2006; Aamidor et al., 2022; Cullen et al., 2022). Despite this similarity, there are differences in the ovarian morphology. While in *B. terrestris* both queens and workers have four ovaries in each ovary, the worker bee ovaries have much fewer ovarioles than those of queens. Queen honeybees have 150–200 ovarioles per ovary, while worker bees have approximately four ovarioles (Snodgrass, 1956; Rozen, 1986; Ronai et al., 2017). During oogenesis, the shape and size ratio of the nurse cells to the oocyte change. In the initial stages of development, the oocyte and the trophocyte follicle increase in size simultaneously. Later the oocyte grows at the expense of the trophocyte. At the end of oogenesis, the oocytes absorbed the trophocytes and reached their final volume. Then the follicle cells form the chorion around the oocyte. The shape of oocytes changes from spherical to oval shape during oogenesis (Duchateau and Velthuis, 1989; Tanaka et al., 2009).

Ovarian growth and oviposition are mainly accompanied by the deposition of vitellogenin, which provides nutrients for later embryonic development. Vitellogenin is synthesized in the fat body and is transferred by endocytosis into the ovaries. It then enters the oocyte through membrane-bound receptors (Engelmann, 1979; Raikhel and Dhadialla, 1992; Du et al., 2019). The fat body comprises two main types of cells: adipocytes and oenocytes. Other types of cells in the insect fat body are urocytes, hemoglobin cells, mycetocytes, and chromatocytes (Roma et al., 2010). The fat body is formed by two parts: 1) a perivisceral fat body surrounding the viscera and filling the abdominal cavity up and 2) a parietal fat body bordering the insect integument (Locke, 1969; Chapman, 2012). In *B. morio*, abdominal part of the fat body surrounding the dorsal vessel forms the hepato-nephrotic system along with pericardial cells and hematocytes (Abdalla and Domingues, 2015). This system, among others, functions as an immune barrier against xenobiotics (Abdalla and Domingues, 2015).

To examine how a change in reproductive strategy when workers of *B. terrestris* start laying eggs affects the morphology and mitotic activity of the ovaries, fat body, and pericardial cells we compared DNA replication activity in these tissues in queens and workers of various ages. DNA replication activity was detected by visualization of the incorporated 5-ethynyl-2′-deoxyuridine (EdU). We found that the rate of DNA replication depended on the age of the individuals and types of tissues tested. Intense DNA replication was detected in the ovaries, fat body, and pericardial cells of 3- to 8-day-old queens. In 1-year-old queens, no replication was observed in the ovaries within 12 h, but fat body cells were mitotically active. In 2- to 3-week-old queens and equally aged non-egg-laying workers with mature ovaries, and in egg-laying workers, DNA synthesis was detected only in the ovaries. DNA replication in nuclei of the nurse cells appeared in differentiated egg chambers indicating a process of endoreplication during vitellogenesis. Endoreplication was synchronous in the proximal vitellarium and progressed only in the nuclei of some nurse cells.

## 2 Materials and methods

### 2.1 Animals

Colonies of the bumblebee, *B. terrestris*, were reared in Koppert Biological System, Nove Zamky, Slovakia. The experimental animals were divided into the following groups: 1) 3- to 8-day-old virgin queens, collected from the colonies 24 h post eclosion; 2) 15- to 20-day-old mated pre-diapause queens, collected from the colonies 24 h after mating at the age of 10-days; 3) 1-year-old, copulated reproducing queens collected 45–48 weeks post diapause termination from colonies in the terminal phase, i.e., after the switch and the competition points; 4) ovipositing workers, collected from 11-week-old queenright colonies after the competition points; 5) workers, maximum 3 weeks old. Colonies were maintained in plastic hives under controlled conditions at 28°C ± 2°C and a relative humidity of 50%–60%. A sugar solution (mixture of glucose, fructose, and sucrose, 48°Brix) and fresh frozen honeybee corbicula pollen were administered *ad libitum* during the rearing process. The animals taken from colonies were kept in plastic nest boxes (7.5 cm × 17.5 cm × 16 cm) with an *ad libitum* supply of sugar solution and bee pollen until they were used for experiments (Koubova et al., 2019). From each group, 4–5 individuals were tested.

### 2.2 Measuring of DNA synthesis

Click-iT EdU Imaging Kits (Invitrogen by Thermo Fisher Scientific, Waltham, MA, United States) were used for directly measuring DNA synthesis in the fat body, pericardial cells, and ovaries of bumblebee castes. The bumblebees were injected with 1–2 µL of 40 mM solution of EdU. After 12 h, the injected bumblebees began to show signs of deteriorating fitness and therefore they were dissected after 12 h. An alternative procedure was tried when EdU was applied to dissected tissue. The selected tissues were dissected in culture media (Sigma-Aldrich Inc., St. Louis, MO, United States) and cultivated for 24 h in M3 insect media supplemented with fetal bovine serum (12%), insulin (0.1%), antibiotic/antimycotic solution (1%), and 0.4 mM EdU solution (i.e., in the concentration that corresponded to the concentration of EdU in the hemolymph when a solution of EdU was injected into an experimental animal). A 24 h incubation of the dissected tissues in solution of EdU did not lead to an increase in the amount of signal, compared to the results obtained after EdU injection into the experimental animals. Presumably, the length of time we can examined DNA synthesis is affected by the stability of the labeled nucleotides. For all reported results, we used samples that were collected from individuals 12 h after EdU injection.

Organs were fixed in 3.7% formaldehyde in phosphate-buffered saline (PBS) for 2 h and then washed in PBS three times for 15 min each at room temperature (RT). The last wash (15 min) was performed in PBS supplemented with 0.5% Triton X-100. Blocking with bovine serum albumin (3% in PBS, 30 min at RT) was followed by incubation with the Click-iT reaction cocktail overnight at 4°C. The reaction cocktail containing Alexa Fluor azide and CuSO_4_ in reaction buffer was prepared according to the manufacturer’s instructions for the Click-iT EdU Imaging Kits. After rinsing with PBS (three times for 15 min at RT), the samples were treated with a solution of 4′-6-diamidino-2-phenylindole (DAPI, 1 µg DAPI/1 mL distilled H_2_O) for 10 min at RT. The reaction was stopped using distilled H_2_O, and the samples were dehydrated in an ethanol series (50%, 70%, 90%, and 100% for 15 min each). A volume of methyl salicylate equal to that of 100% ethanol was added. After evaporation of ethanol, the tissues were stored and mounted in methyl-salicylate. Labelled cells were visualized under a FluoView FV1000 confocal laser scanning microscope (Olympus, Japan) using the UPLSAPO ×10 objective, with correction of brightness in depth and multilocational scanning. Images of the whole ovary were reconstructed by stitching frames using XuvStitch software (XuvTools).

### 2.3 Staining of lipids and actine filaments

For visualization of neutral lipids in the ovaries, staining with 4,4-Difluoro-1,3,5,7,8-Pentamethyl-4-Bora-3a,4a-Diaza-s-Indacene (BODIPY 493/503, Invitrogen) was used. Actin filaments were labeled with phalloidin (Phalloidine-Atto 565, Sigma-Aldrich). Ovariole was dissected in Ringer’s solution and fixed in 3.7% formaldehyde in PBS for 2 h, washed in PBS (3 times for 10 min) and stained with BODIPY and phalloidine according to the manufacture protocols. After washing in PBS (3 times for 10 min), tissues were mounted in Fluoreshield with DAPI (Sigma-Aldrich). The fat body from all over the body was dissected in Ringer’s solution and fixed in 3.7% formaldehyde in PBS for 2 h. The tissues were rinsed with 60% isopropanol and stained with Oil Red O solution (0.5% in isopropanol) for 10 min at RT. After rinsing with 60% isopropanol and PBS, the samples were mounted in 80% glycerol solution. The stained tissues were visualized under a BX51 brightfield microscope (Olympus) and the FluoView FV1000 laser scanning confocal microscope (Olympus).

## 3 Results

### 3.1 Morphology of *B. terrestris* ovaries

**FIGURE 1:**
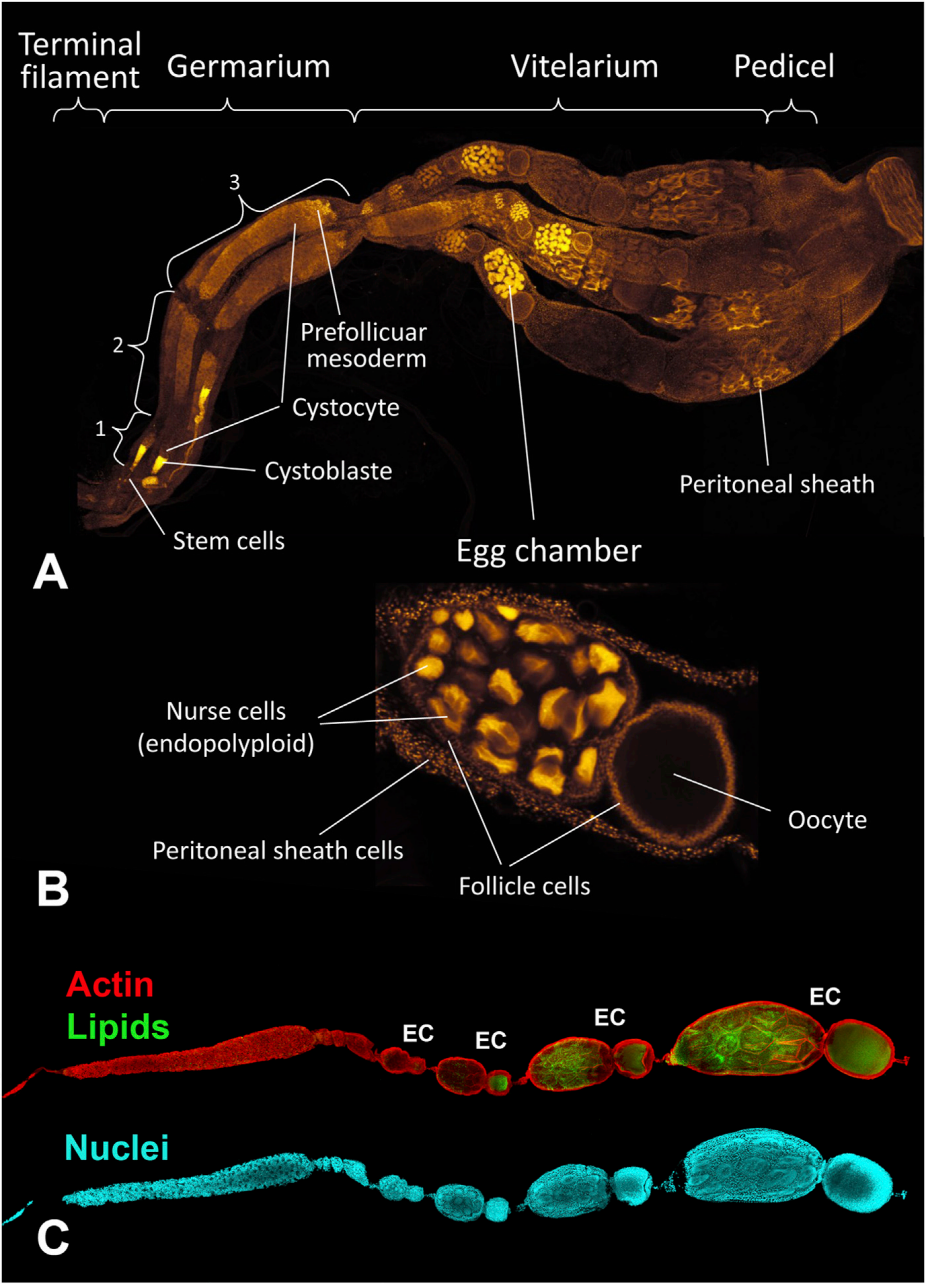
The *B. terrestris* ovary of a 3 day-old unfertilized queen. **(A)** The ovary is formed by four ovarioles, each divided into four parts: the terminal filament, the germarium, the vitellarium, and the pedicel. 1, 2, 3: anterior, middle, and posterior regions of the germarium containing cystoblasts that convert to cystocytes which differentiate into oocytes and nurse cells. The prefollicle mesoderm gives rise to follicle cells. **(B)** An egg chamber surrounded by follicle cells comprises 63 nurse cells with endopolyploid nuclei and an oocyte. Each ovariola is covered by the peritoneal sheath. **(C)** A single ovariole, devoid of the peritoneal sheath with developing egg chambers (EC). Actin filaments are labeled by phalloidin (red), accumulating lipids are stained by BODIPY (green) and nuclei by DAPI (blue).

*B. terrestris* queens and workers have meroistic and polytrophic paired ovaries. Each ovary contains four parallel ovarioles comprising a terminal filament, a germarium, and a vitellarium (Figure 1). The terminal filaments keep the ovary at the right position in the abdomen and contain stem cells. The germarium is clearly divided into three parts: anterior, middle, and posterior regions (no. 1–3 in Figure 1). The anterior part of the germarium possess germ cells, i.e., non-differenced cystoblasts, and the middle and posterior regions of the germarium contain differenced cystocytes that gradually differentiate into oocytes and nurse cells (trophocytes). The prefollicle mesoderm in the most distal part of the germarium gives rise to follicle cells. The follicle cells surround both a group of the 63 nurse cells and an oocyte forming an egg chamber, which matures within the vitellarium. During the maturation process, the nurse part and the oocyte part of an egg chamber become more distinct and gradually increase in size (Figure 1). The entire ovariole is covered by peritoneal sheath cells and is connected by a pedicel to the calyx of a lateral oviduct.

**FIGURE 2:**
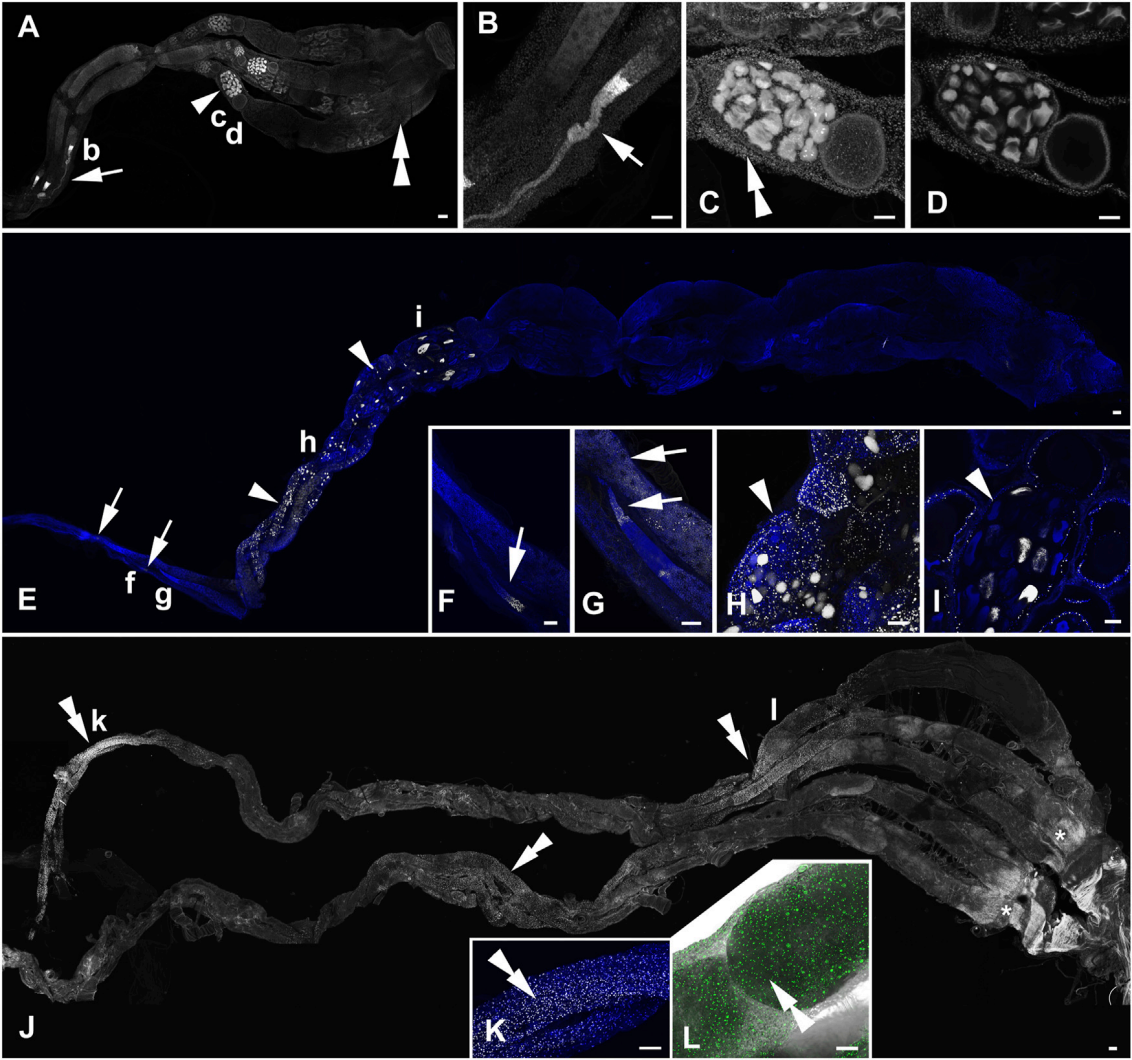
Active DNA synthesis in the ovaries of *B. terrestris* queens within a 12 h period. **(A–D)** The ovary of a representative of 3 to 8-day-old virgin queens. The 3D reconstruction of the whole ovary **(A)**, strong EdU-labelling in the terminal filament and the anterior part of germarium **(B)**, the close-up view of an oocyte chamber with EdU-positive signals in nurse cells (trophocytes), follicle cells, and in the peritoneal sheath of the vitellarium in the Z stack reconstruction **(C)**, and in a single confocal layer **(D)**. **(E–I)** The ovary of a representative of 15- to 20-day-old mated pre diapause queens. Mitotically active cells in the germarium and the vitellarium in the 3D reconstruction of the whole ovary **(E)**, details of the EdU-signal in the anterior part of the germarium **(F,G)**, the close-up view of EdU signals in follicle cells and some trophocytes in an egg chamber of the middle part of the vitellarium in the Z stack reconstruction **(H)** and in a single confocal layer **(I)**. **(J–L)** The ovary of a representative of 1-year-old queens. EdU labelling of some cells of the peritoneal sheath in the 3D reconstruction **(J)**, details of the proximal part of ovarioles **(K)** and the middle part of an ovariole (L, the fluorescence signal is combined with the transmitted light image). The white **(A–K)** and green colors **(L)** indicate localization of EdU incorporated during active DNA synthesis in the nuclei, and the blue color **(E–I,K)** shows DNA labelled by DAPI. The arrows indicate the EdU signal in germ cells of the germarium. Arrowheads show EdU signals in nurse and follicle cells of the vitellarium. Double arrows indicate positive signals in cells of the peritoneal sheath. Asterisks indicate non-specific fluorescence signals **(J)**. Small letters in the ovary overviews indicate the locations from which the corresponding high-magnification images were taken. The scale bars in **(A,E,J)** 100 µm and in **(B-D,F–I,K,L)** 50 µm.

**FIGURE 3:**
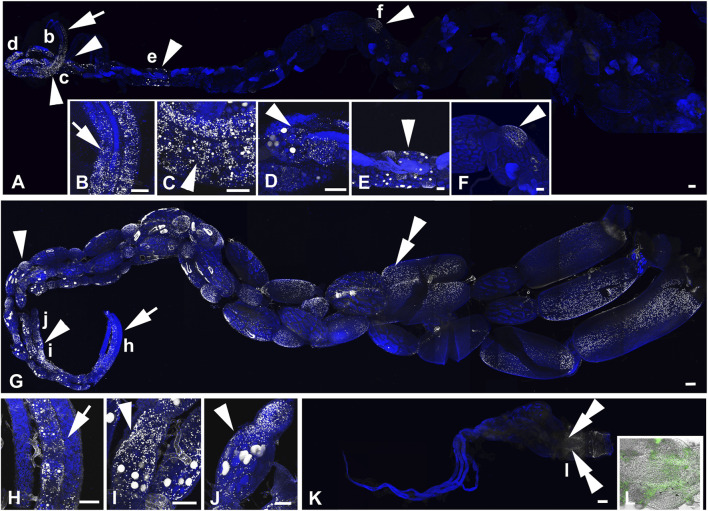
Active DNA synthesis in the ovaries of *B. terrestris* workers within the 12 h period. **(A–F)** The ovary of a representative of ovipositing workers. EdU labelling in the germarium and vitellarium of an egg-laying worker ovary **(A)**, the details of the EdU signal in the germarium **(B)**, close-up view of EdU signals in some trophocytes and follicle cells in the proximal part of the vitellarium **(C–D)**, EdU-signal detection of mitotically active trophocytes and follicle cells in egg chambers of the middle part **(E)** and the distal part **(F)** of the vitellarium. **(G–L)** The ovary of a representative of 1- to 3-week-old workers. The 3D reconstruction of the whole ovary **(G)**. Detailed view of no-EdU-positive germarium of two ovarioles and the vitellarium of an ovary with EdU signals in both nurse and follicle cells **(H)**, details of the EdU signal in some trophocytes and follicle cells of egg chambers **(I–J)**. Topography of a worker with small ovaries with the only EdU signal in some cells in the peritoneal sheath at the distal part of the ovary **(K)**. Details of EdU-positive cells in the peritoneal sheath observed using 3D fluorescence and light confocal images **(L)**. The white **(A–K)** and green colors **(L)** indicate localization of EdU incorporated in active DNA synthesis in the nuclei, and the blue color shows DNA labelled by DAPI. The arrows, arrowheads, and double arrowheads in topography of the whole ovaries show EdU signals detected in the follicle and nurse cells of the germarium, the vitellarium, and cells of the peritoneal sheath, respectively. Small letters in the ovary overviews indicate the locations from which the corresponding high-magnification images were taken. Scale bars in **(A,G,K)** 200 µm and in **(B–F,H–J,L)** 100 µm.

The size ration between the nurse part and the oocyte part of an egg chamber changes during late vitellogenesis so that the nurse part shrinks and disappears completely, while the oocyte becomes larger and longer (Figures 2, 3). The shape of the nucleus of the nurse cells changes from a round shape in the anterior vitellarium to an irregular shape in the middle and posterior regions of the vitellarium. The number of cells in the nurse part of the egg chamber changes during vitellogenesis, so that there were 63 cells with clearly distinguishable round nuclei in the anterior part of the vitellarium. In the middle part of the vitellarium the number of cells decreases (in some selected chambers, 56–48 round shaped nuclei were counted). In the posterior part of the vitellarium, there were only about 30 nurse cells with elongated nuclei of irregular shape (Figures 2E, 3G). In the egg chamber with a mature oocyte, the nuclear DNA of nurse cells was condensed, indicating a possible process of apoptosis (Figure 3G).

The ovaries of 3- to 8-day-old unfertilized queens were 2–3 times smaller than those of both 15- to 20-day-old mated queens and 1-year-old queens (Figure 2). The ovaries of 15- to 20-day-old mated queens were the same size as those of egg-laying workers. Among 1- to 3-week-old workers, individuals with different ovary sizes were observed: 1) mature ovaries with a size comparable to that of egg-laying workers, 2) non-developed ovaries with a size similar to that of 3- to 8-day-old unfertilized queens (Figures 2, 3).

### 3.2 Morphology of the fat body and pericardial cells in *B. terrestris*

**FIGURE 4:**
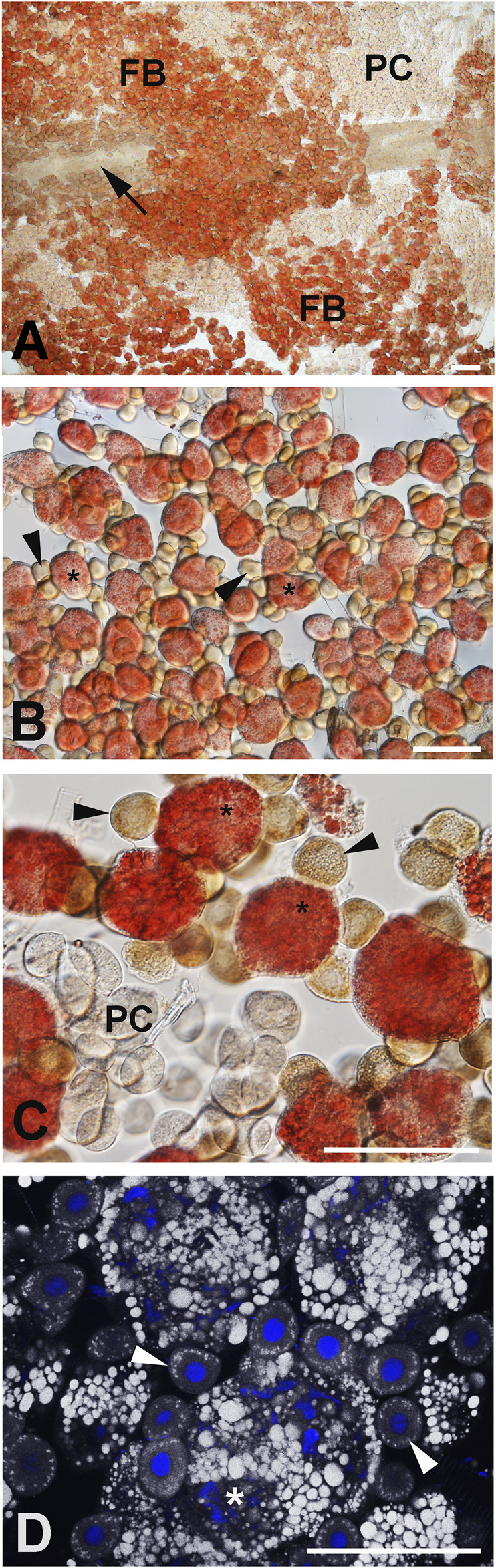
Topography of the abdominal part of the parietal fat body and pericardial cells in a 3-day-old virgin queen. Lipid droplets were visualized by Oil Red O staining and imaged by light **(A–C)** and fluorescence microscopy **(D)**. The pericardial cells (PCs) form an integral strip along the tubular heart (arrow) and the dorsally laid layer of the fat body (FB). Lipid droplets [red in **(B,C)**, and white in **(D)**] in adipocytes (asterisks), oenocytes (arrowheads), and pericardial cells (PC). Nuclei are stained by DAPI (blue) in **(D)**. Scale bars in **(A)** 200 μm, in **(B–D)** 100 µm.

**FIGURE 5:**
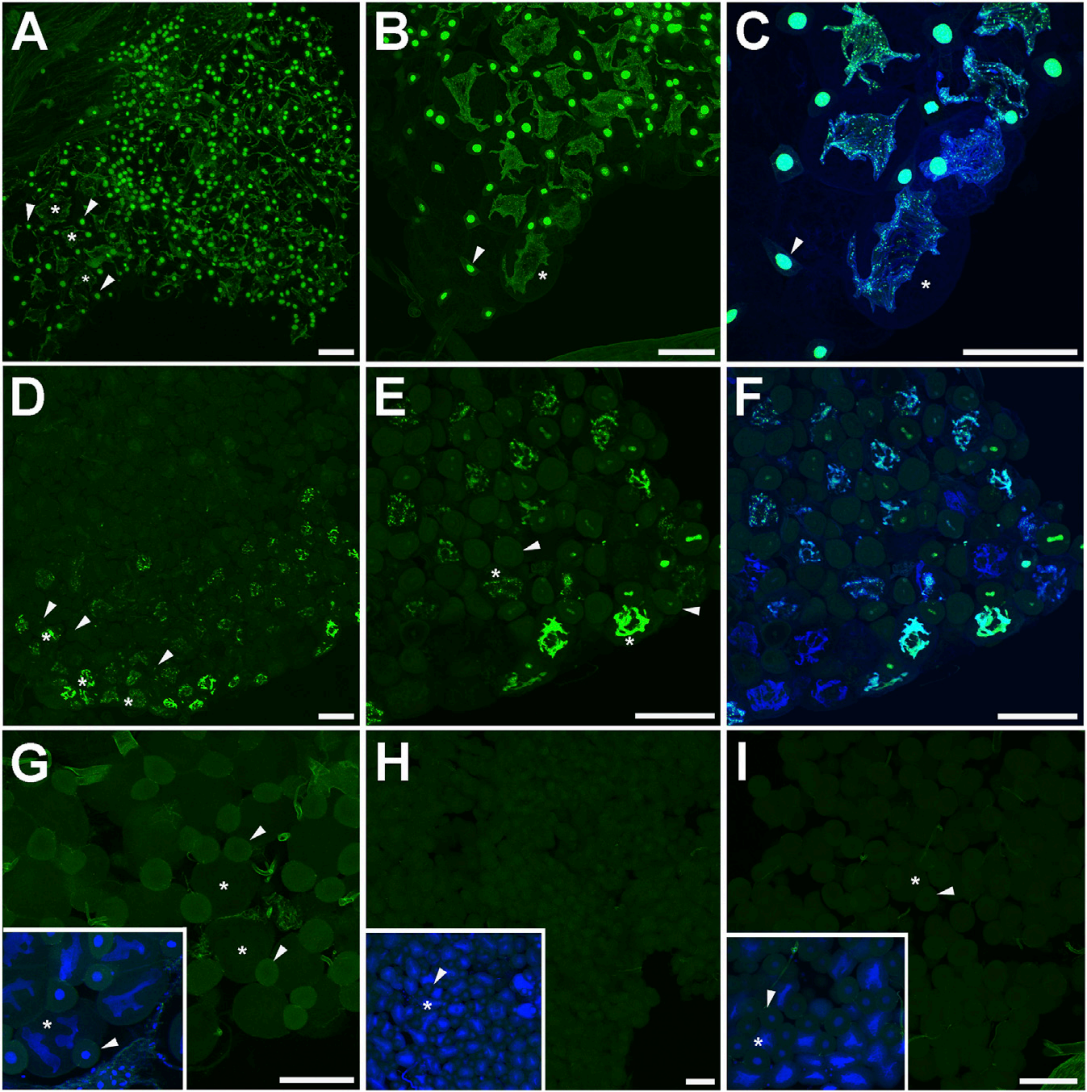
DNA synthesis in the abdominal fat body of bumblebee castes. **(A–C)** The dorsal–lateral part of the parietal fat body of 5- to 10-day-old virgin queens, **(D–F)** 1-year-old queens, **(G)** 15- to 20-day-old mated queens, **(H)** ovipositing workers, and **(I)** 1- to 3-week-old workers. The green color indicates localization of EdU incorporated during active DNA synthesis in nuclei. The green-blue color represents colocalization of EdU signals and DNA labelled by DAPI (blue). Asterisks point to adipocytes, and arrowheads show oenocytes. Scale bar 100 µm.

The fat bodies of *B. terrestris* queens and workers contain two cell types, larger adipocytes and smaller oenocytes (Figures 4, 5). The adipocytes have irregularly shaped nuclei due to numerous large vacuoles filled with lipid droplets. Several oenocytes with round shaped nuclei are attached to the surface of each adipocyte. The cytoplasm of the oenocytes contains small lipid vacuoles. Detailed information regarding cell size and lipid content in different *B. terrestris* castes has been published in a previous study (Koubova et al., 2019).

**FIGURE 6:**
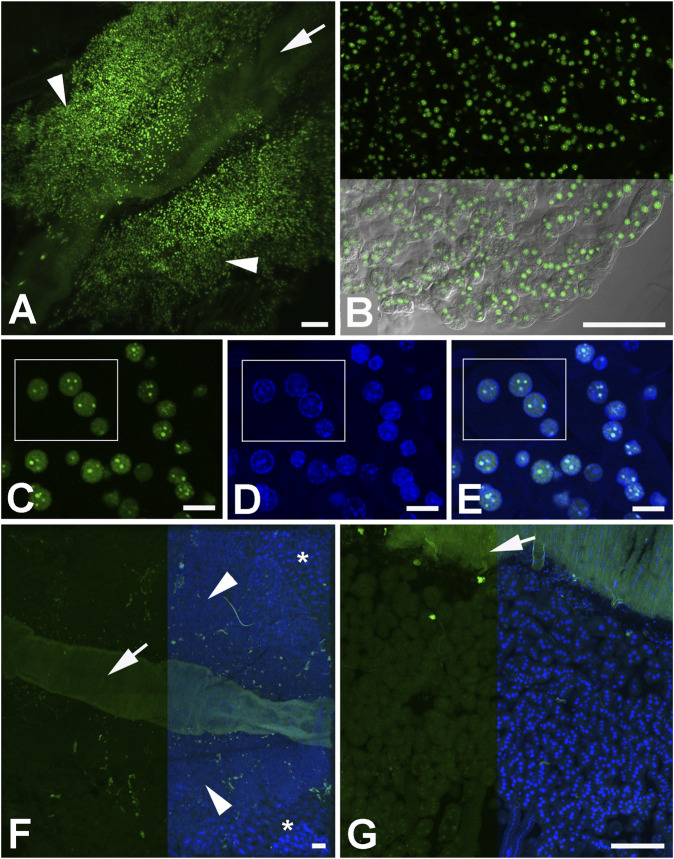
Active DNA synthesis in the pericardial cells of bumblebee castes. **(A–E)** Pericardial cells of 5- to 10-day-old virgin queens. The white rectangle **(C–E)** surrounds a pericardial cell with 4 nuclei. **(F)** Pericardial cells of 1-year-old queens and **(G)** workers. The green color indicates localization of EdU incorporated in active DNA synthesis in nuclei. The green-blue color represents colocalization of EdU signals and DNA labelled by DAPI (blue). Pericardial cells are indicated by arrowheads, the fat body is pointed by asterisks, and the abdominal aorta (heart) by arrows. Scale bars in **(A,B,F,G)** 100 µm and in **(C,D,E)** 10 µm.

Both queens and workers of *B. terrestris* contain numerous pericardial cells that form two bands located laterally from the aorta and are adjacent to the dorsal part of the parietal fat body (Figures 4A, 6A, F, G). Pericardial cells of *B. terrestris* are multinucleate cells, each containing four nuclei (Figures 6B–E).

### 3.3 DNA synthesis in bumblebee ovaries depends on the stage of ovarian maturation, and its distribution indicates the endopolyploidy of nurse cells

Active DNA synthesis during the S-phase of the cell cycle was detected based on the visualization of the incorporated EdU, which is a nucleoside analog of thymidine. EdU contains an alkyne group that reacts with the azide in the Alexa Fluor dye through a copper-catalyzed reaction. DNA synthesis within 12 h was examined in representatives of *B. terrestris* queens and workers: 3- to 8-day-old virgin queens; 15- to 20-day-old mated pre-diapause queens and 1-year-old queens; ovipositing workers; and 1-3-week-old workers (for detailed description of the animals examined, see Material and Methods).

The ovaries of 3- to 8-day-old virgin queens exhibited signals of incorporated EdU in all parts of ovaries (Figures 2A–D). Active DNA synthesis occurred in the nuclei of stem cells, germ cells (cystoblasts), nurse cells, follicle cells, and peritoneal sheath cells. The most prominent signals were observed in the distal part of the terminal filament and in the anterior part of the germarium, indicating high levels of DNA replication during the process of early oogenesis (Figures 2A, B) when stem cells are multiplied and cystoblasts differentiate into cystocytes. The EdU signal in the distal part of the germarium might represent the follicle cells arising from the prefollicle mesoderm. Another intense signal was detected in all nurse cells within several consecutive mature egg chambers in the anterior part of the vitellarium (Figures 2A, C, D) and in some nurse cells in the posterior part of the vitellarium. DNA replication detected in already differentiated nurse cells reflects an endoreplication process in which the M-phase of the cell cycle is skipped, resulting in a mononuclear polyploid cell. Within the vitellarium, DNA also replicated in numerous follicle cells and peritoneal sheath cells. In 15- to 20-day-old mated queens, EdU signals were detected in the germarium prominently in the most anterior part, suggesting everlasting early oogenesis (Figures 2E–G). Compared to virgin queens, in 15- to 20-day-old mated queens, EdU signals were detected in only the subset of nurse cells in the egg chambers exclusively within the anterior part of the vitellarium (Figures 2E, H, I), suggesting asynchrony endoreplication in nurse cells within one egg chamber. In the remaining part of the vitellarium, we did not detect EdU signals in any types of cells (Figure 2E). No DNA replication was detected in 1-year old queens, except that a weak EdU signal was observed in the nuclei of peritoneal sheath cells (Figures 2J–L).

In the ovaries of ovipositing workers (Figures 3A, B), the pattern of EdU signals was identical to that observed in 15- to 20-day-old mated queens. The most prominent signal occurred in the germarium and anterior vitellarium with subsets of trophocytes in the EdU-positive nurse chambers (Figures 3A, D–F). In addition, the ovipositing workers showed active DNA synthesis in follicle cells and in a few nurse cells in some egg chambers within the middle part of the vitellarium (Figures 3A, C–F). Among the examined 1- to 3-week-old workers, animals with large ovaries with active DNA synthesis (Figures 3G–J) and animals with small ovaries with EdU signals only in cells of peritoneal sheath detected at the distal part of the ovary were observed (Figures 3K, L). In 1- to 3-week-old workers with large ovaries, DNA synthesis was even more frequent than in ovipositing workers and mated queens. In the germarium and in the whole vitellarium, EdU signals were detected in numerous follicle cells. Same as in ovipositing workers and mated queens, only some nurse cells passed the S-phase of their cell cycle (Figures 3G–J).

Moderate individual differences in EdU signal distribution and intensity were observed between both ovaries from the same individual. The results observed in the majority of individuals of a given group of experimental animals are presented in the study.

### 3.4 Active DNA synthesis in the fat body and pericardial cells of bumblebee castes

To examine the mitotic activity in the fat body, the fat body was dissected from all over the *B. terrestris* body, including the abdomen, thorax, and head. We examined both parietal and perivisceral fat body. Active DNA synthesis was investigated within a period of 12 h. While in the 3- to 8-day-old virgin queens, most nuclei of adipocytes as well as of oenocytes of both the parietal and perivisceral fat body were mitotically active (Figures 5A–C), only tens of EdU positive cells were observed in the fat body of 1-year-old queens located in the lateral part of the parietal fat body (Figures 5D–F). No active DNA synthesis was detected in the parietal and perivisceral fat body of 15- to 20-day-old mated queens (Figure 5G). Neither ovipositing workers nor representatives of 1- to 3-week-old workers exhibited active DNA synthesis in fat body cells (Figures 5H, I).

During the same time, active DNA synthesis in pericardial cells occurred only in representatives of 3- to 8-day-old virgin queens (Figures 6A–E). EdU signals were localized in all four nuclei of each pericardial cell in clearly distinguishable spots (Figures 6C–E). Pericardial cells of 1-year-old queens (Figure 6F) and 15- to 20-day-old mated queens did not exhibit active DNA synthesis in their nuclei. No EdU signals were detected in pericardial cells in ovipositing workers and 1- to 3-week-old workers (Figure 6G) within the 12 h of examination.

## 4 Discussion

This morphological study of *B. terrestris* ovaries performed using laser scanning confocal microscopy showed that each oocyte in the egg chambers formed in the proximal vitellarium is followed by 63 nurse cells. Later, during oogenesis the number of nurse cells and the shape of their nuclei changed. At the beginning of vitellogenesis, each egg chamber contained 63 nurse cells with a round nucleus. In the middle and posterior regions of the vitellarium, the number of nurse cells decreased to 56–48 cells. There were approximately 30 nurse cells in an egg chamber at the time when the irregularly shaped nuclei were formed. The nuclear DNA of the nurse cells condenses most likely due to the apoptosis in the mature egg chamber. When the oocyte reaches its final volume, the follicle cells form the chorion around the oocyte (Duchateau and Velthuis, 1988).

In *B. terrestris*, as a representative of insect species possessing polytrophic meroistic ovaries, the germarial cystoblast gives rise to a cluster of cystocytes that divide incompletely and remain connected by cytoplasmic bridges that allow the transfer of cytoplasmic macromolecules into the oocyte (i.e., the cystocyte with the largest number of cytoplasmic connections), while the other cystocytes give rise to nurse cells. It has long been assumed that in all insects with polytrophic meroistic ovaries, with some exceptions, the power of 2 rule (2^n^ rule, where *n* equals the number of consecutive mitoses preceding meiosis) applies to cystocytes division (Kerkut and Gilbert, 1985; Hatakeyama et al., 1990; Büning, 1994). However, a recent study showed that this universal rule does not apply in some hymenopteras, including *B. terrestris* (Eastin et al., 2020). The resulting number of cystocytes was a number equal to 2^n^, but it was not generated by *n* times division of the original cystoblast but the earliest formed cystocytes cease to divide during the latter mitotic cycles while their descendants undergo further division (Eastin et al., 2020). In that study, there was identified 63 nurse cells in *B. terrestris* egg chamber during early oogenesis, when the first 8 cystocytes (2 of which were connected to the other cells by five cytoplasmic bridges) were connected to two linear chains of three and four cystocytes, respectively (Eastin et al., 2020). In our study we also identifed 63 nurse cells during early oogenesis. To reduce the number of nurse cells down to 30 cells in the posterior part of the ovary is associated with significant endoreduplication of the nuclei of the remaining cells, which will ensure a sufficient source of mRNA and proteins for the maturing oocyte. In another study (Hopkins and King, 1966), there was reported only 48 nurse cells in *B. terrestris* egg chamber. Authors most likely investigated egg chambers in the later stages of the vitellogenesis.

DNA replication rates over 12 h were studied in queens of different ages and in egg-laying and young workers of *B. terrestris*. The DNA replication rates in stem cells, germ cells, nurse cells, and follicle cells were high in the ovaries of unfertilized queens aged 3–8 days old during the reproductive, previtellogenic, and vitellogenic phases of oogenesis. In the several consecutive egg chambers in the anterior vitellarium, all 63 determined nurse cells undergo DNA endoreplication at least once within 12 h forming endopolyploid cells. In the middle and posterior vitellarium, only some endopolyploid cells performed mitotic activity, indicating that endoreplication in the nurse cells does not proceed synchronously during the entire process of vitellogenesis. The ongoing endoreplication is also evidenced by the changes in the shape of the nucleus of the nurse cells from a round shape in the anterior vitellarium to an irregular shape in the middle and posterior regions of the vitellarium. The process of endoreplication based on the presence of mitotic activity and irregular nuclear shape was suggested to occur in the ovaries of the stingless bee *Melipona quadrifasciata* (Tanaka et al., 2009). Endoreplication during vitellogenesis was studied in detail in the fruit fly *Drosophila melanogaster* (Wilde and Loof, 1973; Koryakov et al., 2013; Ali-Murthy et al., 2020) and the Mediterranean flour moth *Ephestia kuehniella* (Traut et al., 2019). Various stages of the endomitotic cycle with different degrees of chromatin compaction in the nuclei of nurse cells indicating their asynchronous endoreplication have been described in the blow fly *Calliphora erythrocephala* Mg. (Anan’ina et al., 2005).

The ovaries of 3- to 8-day-old pre-diapause virgin queens show significant levels of DNA synthesis. The ovaries of *B. terrestris* queens remain undeveloped even after mating in autumn (Sarro et al., 2022). After overwintering and diapause termination in spring, ovarian maturation continues due the increased level of juvenile hormone, which has a gonadotrophic effect (Larrere and Couillaud, 1993; Larrere et al., 1993; Heinrich, 2004). The process of reproduction and oviposition is accelerated after the first egg is laid, when the presence of workers positively affects the level of juvenile hormone in the queen and the development of her ovaries (Shpigler et al., 2014; Woodard et al., 2014; Sarro et al., 2021; Sarro et al., 2022). Our results show that active oogenesis is already occurring in young unfertilized queens, although the ovaries are small and are not yet fully developed. The number of DNA replications in the ovaries of 15- to 20-day-old mated pre-diapause queens is lower than that in the 3- to 8-day-old queens. Within a single egg chamber in the anterior region of vitellarium of 15- to 20-day-old mated queens, only some nurse cells and follicle cells were labelled. In the 1-year-old queens, no DNA replication was detected in oogonia, nurse cells, or follicle cells. Queens of this age either no longer lay eggs or eggs resorption occurs. Resorption of mature eggs is very common not only in queens but also in workers (Duchateau and Velthuis, 1988).

The ovaries of egg-laying workers from an 11-week-old colony have comparable levels of DNA synthesis to the ovaries of 15- to 20-day-old mated pre diapause queens, both in terms of distribution and the intensity of the EdU signal. Among 1- to 3-week-old workers, individuals with ovaries at different stages of development both mature and non-developed ovaries were found. Their developmental stage corresponded to their size (mature ovaries were 2–3 times larger than undeveloped ones) and was positively correlated with the body size of individual workers. The oogonia and nurse cells of mature ovaries of 1- to 3-week-old workers showed mitotic activity in similar regions of the ovaries as observed in 15- to 20-day-old mated queens. Concerning mature ovary follicular cells in 1- to 3-week-old workers, intense DNA replication activity was observed within all parts of the ovaria. The 1- to 3-week-old workers with non-developed ovaries showed no DNA replication. Since the size of ovaries and their DNA replication activity in mated pre-diapause queens are similar to those of egg-laying workers and non-egg-laying workers with mature ovaries, the dynamics of ovarian activity depends more on the rate of maturation of ovaries and is relatively independent of caste. A similar phenomenon was found in the stingless bee *Melipona quadrifascita*, where the mitotic activity of ovaries detected using 5-bromo-2′deoxy-uridine (BrdU) immunohistochemistry in young queens and workers was similar, and oogenesis in workers was found to depend on age rather than on social interactions (Tanaka et al., 2009).

*B. terrestris* workers of the same age can have ovaries at different stages of development. The development of oviposition in workers begins on average ages of 9–15 days (Duchateau and Velthuis, 1989; Gosterit et al., 2016), and oviposition can occur between 6 and 81 days of age (Bloch and Hefetz, 1999). The ovaries of some workers may contain mature eggs several weeks before oviposition begins, and resorption occurs if they are not egg-laying (Roseler, 1974b). Oviposition development can be affected by numerous factors such as social interactions between workers and a queen or between workers (Honk et al., 1981a; Honk et al., 1981b; Roseler et al., 1981; Duchateau and Velthuis, 1989; Bloch and Hefetz, 1999), the size of individuals, fitness and nutrition (Vogt et al., 1998; Vergara et al., 2003; Tanaka et al., 2019), age, and activity of corpora allata (Roseler et al., 1981; Bloch et al., 1996; Cnaani et al., 2000). Previous studies showed that the ovarian activity in older workers is almost the same in both the queenright and queenless groups, but the ovarian activity in young workers aged 5–8 days is lower when a queen is present in the colony (Duchateau and Velthuis, 1989). Young workers in queenright colonies require at least 10 days for their eggs to become fully mature. The presence of the queen prevents the ovipositions of these workers for approximately 31 days. In contrast, workers from queenless colonies may have their eggs mature 5 days after hatching and they can lay eggs from the age of 7 days onwards (Roseler, 1974a; Duchateau and Velthuis, 1989).

The correlation between the size of *B. terrestris* workers and the development of their ovaries depends on the composition of the groups tested. In queenless colonies, the body size of the workers has no influence in determining which workers will develop mature eggs and which will not (Duchateau and Velthuis, 1989). In captive queenright colonies (Honk et al., 1981b), the size of the workers from the first batch of eggs affects their ability to lay eggs. Larger-sized workers have priority in interactions with the queen and do not participate in foraging activities but have the privilege of laying eggs. These workers compete with the queen for the opportunity to lay eggs and eventually push the queen off. Egg-laying workers inhibit oogenesis in younger workers (Honk et al., 1981b). Positive correlations between the weight and ovarian development have been further described in *B. terrestris* workers kept in two types of laboratory groups: one composed of a queen and 8 workers from the first batch and the second consisting of only 8 from the first batch workers without a queen (Gosterit et al., 2016). In groups containing a worker from the first batch with or without a queen, no correlation between weight and ovary development was observed (Gosterit et al., 2016).

Ovarian maturation is also related to the immune system (Sarro et al., 2022). Ovary development can be directly affected by the infestation parasites such as the nematode *Sphaerularia bombi* (Colgan et al., 2020) or indirectly by the fungal parasites *Vairimorpha bombi* and *Apicystis bombi* through negative effects on fat body reserves and survival (Schmid-Hempel, 2001; Mullins et al., 2020). The hepato-nephrotic system is important for protection against these parasites and other environmental stressors such as xenobiotics. The components of the hepato-nephrotic system are the fat body, hemocytes, and pericardial cells, which act in synergy to form an effective barrier (Abdalla and Domingues, 2015). Pericardial cells exhibit pinocytic and phagocytic activities, have an excretory function, and participate in the absorption of toxic substances (Fife et al., 1987; Poiani and da Cruz-Landim, 2007).

In virgin queens, high DNA synthesis was observed in both types of fat body cells (adipocytes and oenocytes) and in pericardial cells. This mitotic activity may reflect the ongoing cell proliferation of fat body and pericardial cells, which is related to the development of the hepato-nephrotic system. Because no DNA replication was detected in mated pre-diapause queens and in both ovipositing and non-egg-laying workers during the 12 h studied, the division of the fat body and pericardial cells may have stopped or occurred at a lower frequency at this stage of life. These results are correlated with evidence of strong upregulation of DNA synthesis in the fat body during early age of pre-diapause *B. terrestris* queens and workers (Koubova et al., 2019). In 1-year-old queens, mitotic activity occurred only in certain parts of the fat body collected from the thorax and abdomen, and no DNA synthesis was observed in pericardial cells. Fat body formation is also related to metabolism and various biochemical processes such as vitellogenin production (Raikhel and Dhadialla, 1992; Du et al., 2019). In insects, vitellogenin production in fat body cells is controlled by the juvenile hormone (Wu et al., 2020). Vitellogenin is transported to the oocytes via membrane-bound receptors that not only mediate the transport of vitellogenin but also play an important role in vitellogenesis (Du et al., 2019). Low transcript levels of vitellogenin were observed in virgin queens in which most of the nuclei of the fat body and pericardial cells were in their S-phase. The high transcript level of vitellogenin was observed in oviposition workers and in 1-year-old queens (Jedlicka et al., 2016; Koubova et al., 2019).

## 5 Conclusion

We examined ovarian mitotic activity in *B. terrestris* females during oogenesis. At the beginning of the vitellogenin phase, an egg chamber in the ovaries of both queens and workers contains 63 nurse cells and one oocyte, which are surrounded by follicular cells. The nurse cells, separated by the follicular cells from the oocyte, undergo endoreplication. During vitellogenesis, their endoreplication becomes asynchronous and the number of nurse cells decreases. The initial phase of the ovarian maturation (3- to 8-day-old virgin queens) is characterized by intense mitotic activity, and during the 12 h period, most cells of the germarium and vitellarium pass through the S-phase of their cell cycles. DNA synthesis observed during the 12 h period in mated pre-diapause queens and ovipositing and non-egg-laying workers with mature ovaries was restricted to the germarium and the anterior part of the vitellarium, whereas in 1-year-old queens, replication occurs only in the peritoneal sheath cells during the same time interval. The rate of replication activity in ovaries correlates with the rate of the ovarian maturation rather than the caste membership.

## Data Availability

The raw data supporting the conclusion of this article will be made available by the authors, without undue reservation.
